# Macrophage Migration Inhibitor Factor Upregulates MCP-1 Expression in an Autocrine Manner in Hepatocytes during Acute Mouse Liver Injury

**DOI:** 10.1038/srep27665

**Published:** 2016-06-08

**Authors:** Jieshi Xie, Le Yang, Lei Tian, Weiyang Li, Lin Yang, Liying Li

**Affiliations:** 1 Department of Cell Biology, Municipal Laboratory for Liver Protection and Regulation of Regeneration, Capital Medical University, Beijing 100069, China

## Abstract

Macrophage migration inhibitor factor (MIF), a multipotent innate immune mediator, is an upstream component of the inflammatory cascade in diseases such as liver disease. Monocyte chemoattractant protein-1 (MCP-1), a highly representative chemokine, is critical in liver disease pathogenesis. We investigated the role of MIF in regulating hepatocytic MCP-1 expression. MIF and MCP-1 expression were characterized by immunochemistry, RT-PCR, ELISA, and immunoblotting in CCl_4_-treated mouse liver and isolated hepatocytes. MIF was primarily distributed in hepatocytes, and its expression increased upon acute liver injury. Its expression was also increased in injured hepatocytes, induced by LPS or CCl_4_, which mimic liver injury *in vitro*. MIF was expressed earlier than MCP-1, strongly inducing hepatocytic MCP-1 expression. Moreover, the increase in MCP-1 expression induced by MIF was inhibited by CD74- or CD44-specific siRNAs and SB203580, a p38 MAPK inhibitor. Further, CD74 or CD44 deficiency effectively inhibited MIF-induced p38 activation. MIF inhibitor ISO-1 reduced MCP-1 expression and p38 phosphorylation in CCl_4_-treated mouse liver. Our results showed that MIF regulates MCP-1 expression in hepatocytes of injured liver via CD74, CD44, and p38 MAPK in an autocrine manner, providing compelling information on the role of MIF in liver injury, and implying a new regulatory mechanism for liver inflammation.

Liver is mainly composed of hepatocytes (~80%), which execute multiple physiological functions^1^,^2^. Liver injury, usually induced by heavy alcohol consumption, drug abuse, and virus infection, is a common clinical disease characterized by hepatocyte injury^3^,^4^,^5^. Recently, many studies have shown that injured hepatocytes can secrete important cytokines and chemokines that are involved in the immune response, by activating and recruiting immunocytes^2^,^6^,^7^. Although the cytokines and chemokines secreted by injured hepatocytes have been considered to participate in liver injury^6^, the molecular mechanism of how these cytokines and chemokines are produced from injured hepatocytes is still indistinct.

Macrophage migration inhibitor factor (MIF), named as such because of its ability to prevent random migration of macrophages, is an important cytokine, and was described almost 50 years ago^8^,^9^. Since its discovery, MIF has been shown to be a multipotent innate immune mediator^10^. For instance, Barnes *et al.*^3^ revealed that MIF played a critical role in monocyte/macrophage recruitment in ethanol-induced liver injury^3^. Additionally, MIF is necessary for neutrophil infiltration in lipopolysaccharide (LPS)-induced acute lung injury in rats^11^. Furthermore, recent studies have reported that MIF can promote the expression of many other cytokines^10^,^12^. Akoum *et al.* indicated that MIF could induce increases in interleukin (IL)-6, IL-8 and PGE2 release from chondrocytes^12^, and IL-8 and MCP-1 could be regulated by MIF in human ectopic endometrial stromal cells^13^. Furthermore, many researches have shown that MIF is produced by multiple cells in liver, such as hepatocytes, endothelial cells, monocytes, and macrophages^10^,^14^,^15^. In particular, hepatocytes, the predominant liver cells, act as the major producer of MIF in autoimmune hepatitis and liver fibrosis^16^,^17^.

The classical receptor of MIF is CD74, which is a membrane-expressed form of the invariant chain and is an MHC class II chaperone^9^. CD74, in spite of its high affinity to MIF, requires coreceptor CD44 to mediate the biological function of MIF^9^,^18^. CD74, in combination with CD44, has been linked to proinflammatory functions of MIF by activation of MAPKs^18^. Ortiz *et al.* reported that CD74 mediates the actions of MIF on inflammatory mediators, TNF-related apoptosis-inducing ligand (TRAIL), and MCP-1, via p38 MAPK in podocytes^19^. CXCR2 and CXCR4 also act as coreceptors of CD74, whereas CD74/CXCR2 and CD74/CXCR4 complexes are more inclined to contribute to MIF-mediated monocyte chemotaxis^9^.

MCP-1, also known as CCL2, belongs to the largest family of chemokines, the CC chemokine family^20^. MCP-1 is one of the most representative chemokines, and is the major determinant of monocyte/macrophage recruitment to the site of tissue injury^21^. A series of reports indicate a critical role of MCP-1 in the pathogenesis of liver disease. Previous researches showed that both MCP-1 antagonism by mNOX-E36 and MCP-1 deficiency could efficiently protect mice against carbon tetrachloride (CCl_4_)-induced acute liver injury^22^,^23^. MCP-1 was also shown to promote steatohepatitis in rodent models of alcoholic steatohepatitis^24^. MCP-1 is released by a large variety of cells such as epithelial cells, monocytes, endothelial cells and smooth muscle cells; and its expression is upregulated following proinflammatory stimulation and tissue injury^24^,^25^. A recent study reported that hepatocytes showed a significant increase in MCP-1 at the mRNA level in ethanol-treated HIV transgenic rats^26^. Additionally, protein levels of MCP-1 were significantly enhanced in hepatocytes treated with hypoxia-mimicking agents^27^.

MIF and MCP-1 levels are positively correlated in patients with chronic hepatitis B^28^. Therefore, in our present study we focused on the relationship between the expression of MIF and MCP-1 in injured hepatocytes. We first expound that the MCP-1 expression in injured hepatocytes is regulated by MIF in an autocrine fashion. Next, we confirmed that MIF executes its regulatory function via CD74, CD44, and the p38 MAPK pathway.

## Results

### Hepatocytic MIF expression was upregulated upon mouse acute liver injury

**Figure 1:**
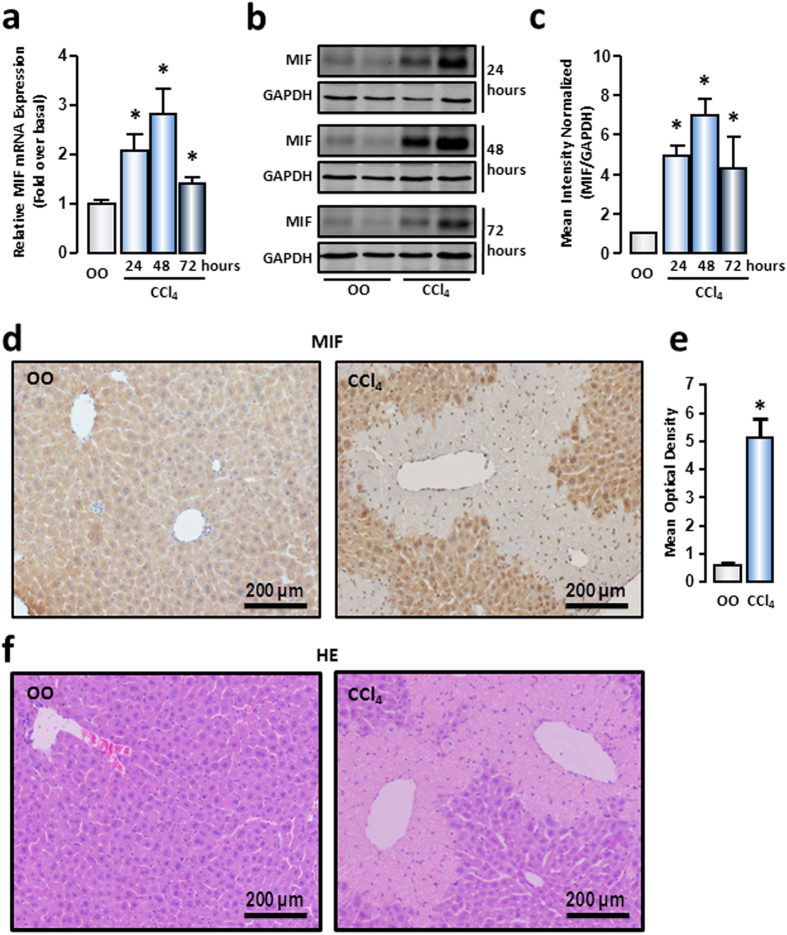
MIF expression and distribution in acute injured liver induced by CCl_4_. After CCl_4_ administration at 24 hours, 48 hours and 72 hours, mRNA (**a**) and protein (**b**) expression of MIF in mice livers were measured by real-time RT-PCR and Western blot, respectively. (**c**) The quantitative assay of Western blot was executed by Odyssey® software. The representative images of immunohistochemical staining (**d**) and HE staining (**f**) were shown to track MIF (brown) in liver tissue of normal or CCl_4_–treated mice. (**e**) The mean optical density in immunohistochemical staining images of MIF was measured by Image-Pro Plus software. Scale bars: 200 μm. Data are presented as the means ± SEM. *P < 0.05 *vs* control group (n = 6, per group).

We assessed MIF content in liver tissue of the mouse acute liver injury model, which was induced by intraperitoneal injection of CCl_4_ (1 μL/g body weight). Results of real-time reverse transcription polymerase chain reaction (RT-PCR) showed that MIF mRNA was significantly upregulated from 24 hours of CCl_4_ administration, with a maximal increase at 48 hours, which then dropped to basal levels at 72 hours (Fig. 1a). To further clarify MIF protein expression in liver tissue, Western blot was performed. Consistent with the mRNA results, the MIF protein content in injured liver tissue also displayed a marked increase at 24 hours and reached maximum level at 48 hours, which came back down at 72 hours (Fig. 1b,c). These results implied that MIF plays a role in the initial phase of liver injury.

In liver, hepatocytes, macrophages, monocytes, and endothelial cells are considered to contribute to MIF production^10^,^14^,^15^. To identify the most important cellular source of MIF in acute liver injury, we performed immunohistochemical assays on liver paraffin sections. According to the images, MIF was shallow-stained in normal liver and primarily existed in hepatocytes, which formed the principal liver cell population. Moreover, the residual hepatocytes were the main producers of MIF in the injured liver. Notably, the intensity of MIF staining in injured liver was much stronger than that in normal liver (Fig. 1d,e). By morphological analysis of H&E-stained liver paraffin sections, we could further confirm that hepatocytes are the primary producers of MIF in normal and injured liver (Fig. 1f). These results illustrated that hepatocytes were the major source of MIF in liver, and increased MIF in injured hepatocytes might play a critical role in liver injury.

### MIF was expressed earlier than MCP-1 in injured hepatocytes *in vitro*

**Figure 2:**
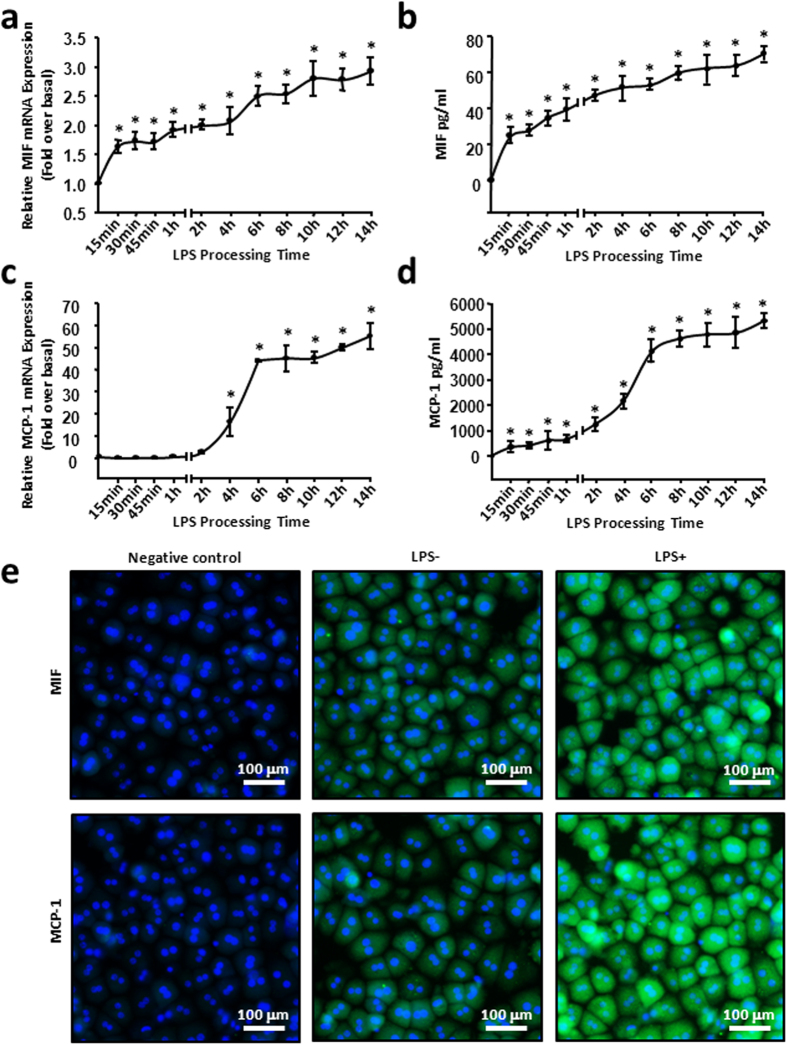
LPS induced increase of MIF appeared earlier than MCP-1 in hepatocytes. Mouse primary haptocytes were treated with 100 ng/mL LPS and collected at the described time points, the relative mRNA (**a,c**) expression and protein secretion (**b,d**) of MIF and MCP-1 were examined by real-time RT-PCR and ELISA. (**e**) The representative images of immunofluorescence for MIF or MCP-1 (green) in mouse primary hepatocytes, as visualized by immunocytochemical analysis. Cells were co-stained with DAPI to identify nuclei (blue). Scale bars, 100 μm. All results were confirmed in three independent experiments at least. *P < 0.05 *vs* untreated control cells.

To identify the function of MIF produced by hepatocytes, we prepared mouse primary hepatocytes from adult mice by liver perfusion. The isolated mouse primary hepatocytes were treated with 100 ng/mL LPS and were collected at various time points as described (0, 15, 30, 45 minutes and 1, 2, 4, 6, 8, 10, 12, 14 hours). MIF mRNA and protein expression was detected. As shown in Fig. 2a, the mRNA level of MIF showed a significant initial increase (1.7-fold at 15 minutes after LPS treatment), then slowly increased in a time-dependent manner, and further increased to 3-fold at 14 hours. To verify the protein expression of MIF in LPS-stimulated hepatocytes, enzyme-linked immunosorbent assay (ELISA) was performed to evaluate the protein content of MIF in hepatocyte culture supernatants. As expected, the content of MIF protein secreted from hepatocytes rapidly increased to ~20 pg/mL after 15 minutes of LPS treatment, and then further increased gradually. The increase lasted for 14 hours, with a maximal content of 70 pg/mL (Fig. 2b). As previous studies had verified that hepatocytes also expressed MCP-1, the most important inflammatory chemokine associated with macrophage infiltration^26^,^27^, we tested the mRNA and protein expression of MCP-1 in injured hepatocytes. In contrast to the change in MIF expression, MCP-1 mRNA expression maintained a low level until 4 hours of LPS treatment, and achieved a plateau at 6 hours, with the increase amounting to approximately 40-fold. The maximal increase of MCP-1 mRNA expression appeared at 14 hours, which was more than 50-fold (Fig. 2c). Secreted MCP-1 protein from injured hepatocytes was also detected by ELISA. Similar to its mRNA expression, secretion of the MCP-1 protein lentamente increased from 15 minutes to 4 hours, reached a plateau of approximately 4000 pg/mL at 6 hours after LPS challenge, then peaked at 14 hours and the maximum content was more than 5000 pg/mL (Fig. 2d). Notably, the amount of MCP-1 protein was much more than that of MIF throughout the experimental period (Fig. 2b,d). Immunofluorescent staining further confirmed that the expression of MIF and MCP-1 markedly increased in hepatocytes under 6 hours of LPS treatment (Fig. 2e). We also observed that the amount of macrophages (F4/80+ cells) were increased markedly in CCl_4_-treated liver, compared with that in normal live (Supplementary Fig. 1a,b), which is consistent with published literature. In addition, the same experimental conditions were applied to AML-12 cells, which are reconstructive, immortalized mouse hepatocytes, and we obtained the same results as with the mouse primary hepatocytes (Supplementary Fig. 2a–e). These results showed that the expression of MIF and MCP-1 were increased in injured hepatocytes *in vitro*, and MIF expression appeared earlier than MCP-1.

### MIF regulated MCP-1 expression in injured hepatocytes in an autocrine manner

**Figure 3:**
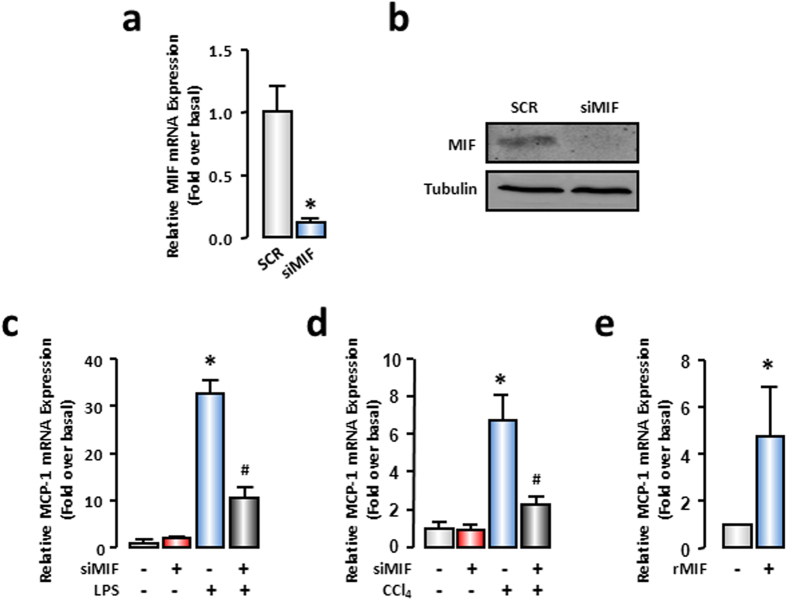
MIF promoted up-regulation of MCP-1 in hepatocytes. AML-12 cells were transfected with SCR siRNAs or MIF siRNAs. 48 hours later, cells were treated with LPS for another 6 hours or CCl_4_ for another 3 hours. The mRNA (**a**) and protein (**b**) expressions of MIF were detected to confirm the efficiency of MIF knockdown. (**c,d**) MCP-1 mRNA expression were evaluated by real-time RT-PCR. (**e**) AML-12 cells were treated by 100 ng/mL mouse re-combinational MIF (rMIF) for 6 hours. MCP-1 mRNA expression were detected by real-time RT-PCR. All results were confirmed in three independent experiments at least. *P < 0.05 *vs* untreated control cells. ^#^*P* < 0.05 *vs* LPS or CCl_4_ treated cells alone.

Next, we explored whether MIF was involved in the regulation of MCP-1 expression in injured hepatocytes. We employed MIF-specific siRNAs and investigated the expression of MCP-1. Transfection of MIF-siRNA decreased the mRNA expression of MIF by 90% in AML-12 cells. The knockdown efficiency satisfied the requirement of the RNAi experiment (Fig. 3a). Meanwhile, Western blot analysis indicated that MIF protein expression was also significantly reduced after MIF knockdown (Fig. 3b). According to the earlier results (Fig. 2c,d), the expression of MCP-1 was dramatically increased at 6 hours after LPS treatment. Thus, in the following experiments, we determined 6 hours as the detection time for LPS stimulation. The deficiency in MIF significantly decreased MCP-1 mRNA expression by ~60% in LPS-treated (100 ng/mL) AML-12, whereas scrambled siRNAs had no effect (Fig. 3c). Another stimulator CCl_4_ (0.5 mM) was used for *in vitro* study to confirm the effect of MIF deficiency on MCP-1 expression, and the results were similar to those in LPS-treated hepatocytes (Fig. 3d). On the other hand, to illustrate the action mode of MIF in regulating MCP-1 expression, we used recombinational MIF (rMIF) to imitate hepatocyte-secreted MIF. The mRNA level of MCP-1 was strikingly increased in the AML-12 cells, trigged by treatment with 100 ng/mL rMIF (Fig. 3e). These results implied that hepatocyte-secreted MIF was the crucial regulator of MCP-1 in injured hepatocytes.

**Figure 4:**
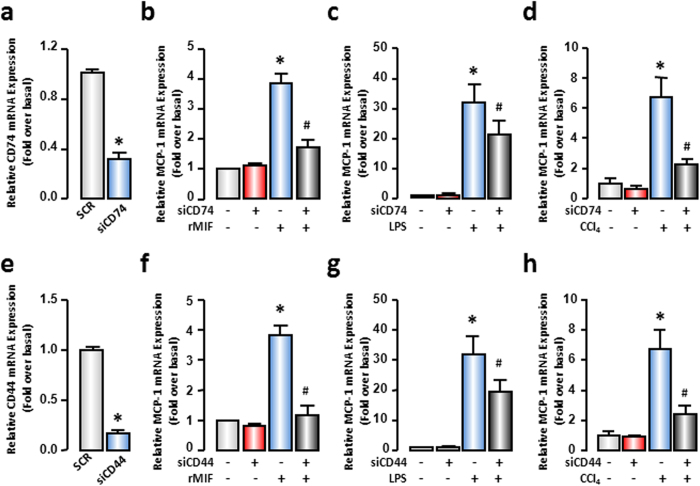
CD74 and CD44 mediated MCP-1 increase in hepatocytes. AML-12 cells were transfected with SCR siRNAs, CD74 siRNAs or CD44 siRNAs. After 48 hours, cells were treated with rMIF or LPS for another 6 hours or CCl_4_ for another 3 hours. The mRNA expressions of CD74 (**a**) and CD44 (**e**) were examined to confirm the knockdown efficiency. MCP-1 mRNA expression were evaluated by real-time RT-PCR (**b–d,f–h**). All results were confirmed in three independent experiments at least. *P < 0.05 *vs* untreated control cells. ^#^*P* < 0.05 *vs* rMIF, LPS or CCl_4_ treated cells alone.

In order to further confirm that the MCP-1 expression is regulated by MIF in an autocrine manner in injured hepatocytes, we employed siRNAs of CD74 and CD44, which are the classical membrane receptors of MIF. After transfection, the mRNA expression of CD74 and CD44 was decreased to ~30% and ~20% of its basal levels, respectively, conforming to the experimental conditions (Fig. 4a,e). Subsequently, MCP-1 mRNA expression was evaluated by real-time RT-PCR in AML-12 cells treated with rMIF (100 ng/mL), LPS (100 ng/mL), or CCl_4_ (0.5 mM) with or without CD74- or CD44-siRNA transfection. As shown in Fig. 4, either CD74 or CD44 knockdown markedly restrained rMIF-induced increases in MCP-1. The MCP-1 mRNA expression was impaired by ~60% and ~70% by CD74- and CD44-siRNAs in several cases (Fig. 4b,f). Additionally, the receptor deficiency inhibited LPS- or CCl_4_-mediated MCP-1 increase. Either CD74 or CD44 deficiency in CCl_4_-injured hepatocytes showed remarkable ability to decrease MCP-1 mRNA expression by ~70% (Fig. 4d,h). However, compared with rMIF-treated hepatocytes, the LPS-stimulated hepatocytes showed a weaker reaction to CD74- or CD44-siRNAs. Both CD74- and CD44-siRNAs caused a decrease in MCP-1 mRNA expression by ~30% (Fig. 4c,g). These results revealed that MIF was one of the important mediators of MCP-1 expression in injured hepatocytes and MIF signaling occurred in an autocrine manner.

### p38 MAPK mediated the effect of hepatocellular MIF on MCP-1 expression in injured hepatocytes

**Figure 5:**
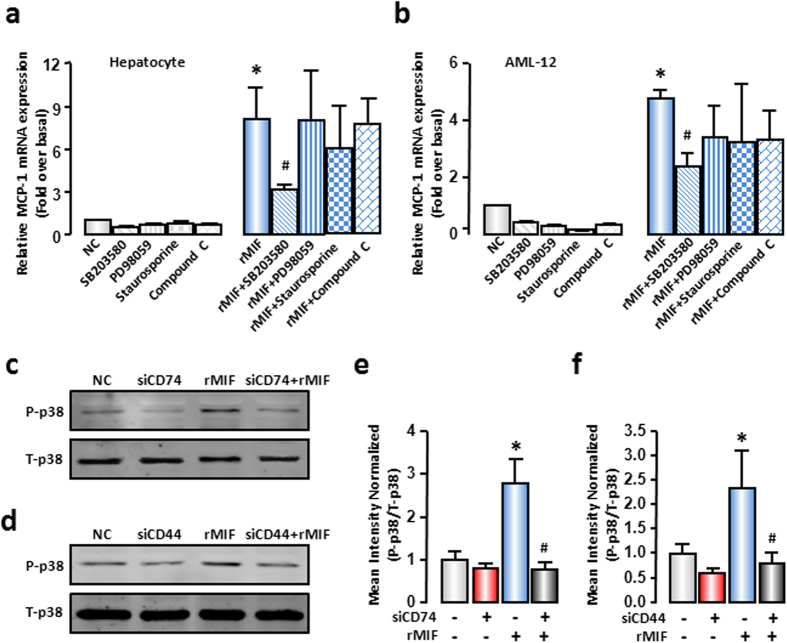
MIF promoted MCP-1 expression through p38 MAPK. (**a**) Mouse primary hepatocytes or (**b**) AML-12 cells were pre-treated with SB203580 (10 μM), PD98059 (10 μM), staurosporine (10 nM), compound C (10 μM) for 1 hour, and followed by 100 ng/mL rMIF treatment for another 6 hours. MCP-1 mRNA expression was evaluated by real-time RT-PCR. (**c,d**) AML-12 cells were transfected with SCR siRNAs, CD74 siRNAs or CD44 siRNAs. After 48 hours, cells were treated with rMIF, after another 6 hours, cells were collected. Total p38 and phosphor-p38 levels were evaluated by Western blot analysis. Typical autoradiograms were shown. (**e,f**) The quantitative assay of Western blot was executed by Odyssey® software. All results were confirmed in three independent experiments at least. *P < 0.05 *vs* untreated control cells. ^#^*P* < 0.05 *vs* rMIF treated cells alone.

The biological function of MIF depends on multiple signaling pathways, such as MAPKs, PKC and AMPK signaling pathways^13^,^29^,^30^. To identify the responsible signal pathway for MCP-1 expression regulated by MIF in hepatocytes, pharmacological inhibitors of signal transduction, SB203580 (p38 inhibitor), PD98059 (ERK inhibitor), staurosporine (PKC inhibitor), and compound C (AMPK inhibitor) were applied. The rMIF-induced increase of MCP-1 mRNA expression was only apparently impaired by SB203580 in mouse primary hepatocytes and AML-12 cells (Fig. 5a,b). Meanwhile, the rMIF alone induced a significant increase in the protein level of phospho-p38 (~2.5 fold), and such changes could be blocked by CD74- or CD44-siRNAs effectively (~0.78 fold and ~0.80 fold, Fig. 5c–f). These results illustrated that p38 activation was involved in MIF-induced MCP-1 expression and MIF-activated p38 through the classical receptors of MIF, CD74, and CD44.

### MIF inhibition alleviated liver MCP-1 expression and p38 phosphorylation in CCl_4_-induced liver injury

**Figure 6:**
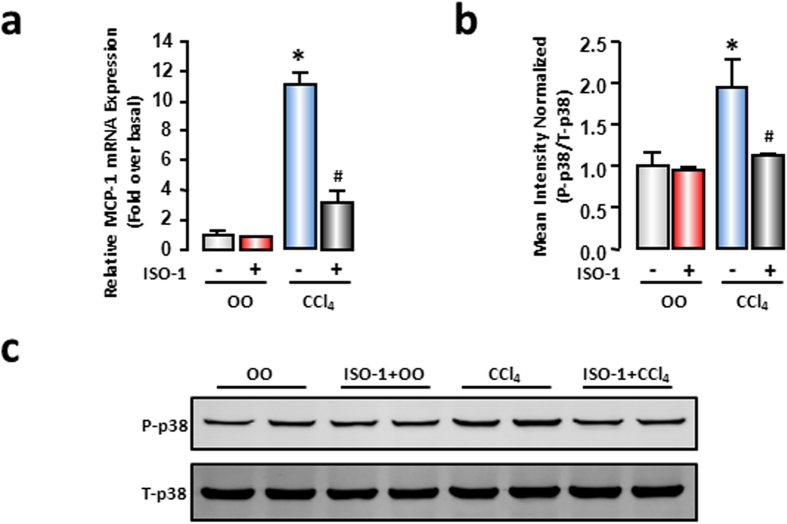
MIF inhibitor (ISO-1) decreased expression of MCP-1 and phosphorylation of p38 in CCl_4_-treated liver. At 15 minutes before CCl_4_ administration, mice received an injection of ISO-1 (35 mg/kg body weight), 48 hours later, liver tissue were collected. (**a**) mRNA expression of MCP-1 were measured by real-time RT-PCR. (**c**) Protein level of total p38 and phosphor-p38 were evaluated by Western blot analysis. (**b**) The quantitative assay of Western blot was executed by Odyssey® software. Data are presented as the means ± SEM. All results were confirmed in three independent experiments at least. *P < 0.05 *vs* control group (n = 6, per group).

To substantiate that MIF regulates MCP-1 expression in autocrine manner *in vivo*, mice suffered from CCl_4_ intraperitoneal injection with or without MIF inhibitor (ISO-1, 35 mg/kg body weight, 15 minutes before CCl_4_ injection, n = 6 per group). Real-time RT-PCR results showed that liver MCP-1 mRNA expression significantly increased after 48 hours of CCl_4_ treatment compared with the vehicle control, and ISO-1 administration relieved the increase of MCP-1 markedly (Fig. 6a). To further identify whether MIF influenced p38 phosphorylation in liver, we detected the phosphorylation level of p38 by Western blot. Phosphorylation of p38 significantly increased in injured liver compared to the vehicle control. Moreover, increased p38 phosphorylation was markedly attenuated by ISO-1 administration (Fig. 6b,c). These results, in accordance with the hepatocyte results, implied that MIF might regulate MCP-1 through the p38 MAPK pathway in an autocrine manner *in vivo*.

## Discussion

**Figure 7:**
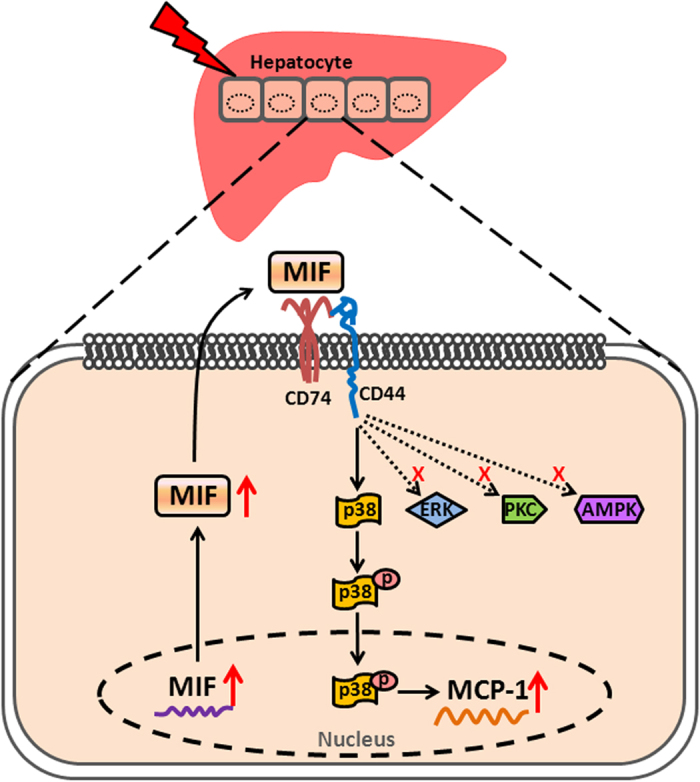
Scheme of MCP-1 expression induced by MIF in hepatocytes. MIF exerts a powerful effect on MCP-1 expression in hepatocytes through CD74 and CD44, which involves the activation of p38 MAPK signaling pathway.

In the current study, we illustrated for the first time that MIF upregulates MCP-1 expression in injured hepatocytes in an autocrine manner. *In vivo*, hepatic MIF expression markedly increased in CCl_4_-induced mouse acute liver-injury models, and was primarily distributed in hepatocytes. *In vitro*, MIF expression was rapidly increased in injured hepatocytes; it was expressed prior to MCP-1. MIF also showed a potential induction of MCP-1 expression in injured hepatocytes. Specific siRNAs against either CD74 or CD44 alleviated the MIF-induced increase in MCP-1 expression. Pretreatment of specific signal-transduction inhibitors further clarified that p38, but not ERK, PKC, or AMPK, mediated the effect of MIF on MCP-1 expression. ISO-1 administration further proved that, *in vivo*, MIF regulated MCP-1 expression through the p38 MAPK pathway. A concept diagram is shown to summarize our major findings (Fig. 7).

Substantive organ inflammatory disease generally involves extensive parenchymal cell injury, which is considered to be involved in regulation of inflammation^6^,^31^,^32^,^33^,^34^. It has been described that hepatocytes are involved in ethanol-induced liver injury by releasing the endogenous danger molecules, uric acid and ATP, which are recognized by liver immune cells^33^. In ischemic-reperfusion-induced kidney injury, nonselective cation channel TRBM2 on renal parenchymal cells could also mediate histologic tissue injury and inflammation by affecting neutrophil infiltration^31^. In our study, we observed that expression of the inflammatory cytokine MIF increased in injured hepatocytes both *in vivo* and *in vitro*, and that increased MIF levels further regulated hepatocellular inflammatory chemokine MCP-1 expression in an autocrine fashion. This is powerful evidence to show that hepatocytes are involved in the liver inflammatory response. This has also been shown by Csak, who reported that hepatocytes exposed to saturated fatty acids release danger signals to trigger inflammasome activation in immune cells in the liver^32^.

Innate inflammation induced by a large degree of innate immunocyte infiltration is a classic symptom of acute liver injury^35^. MCP-1 is one of the most important chemokines, and is the major determinant of recruitment of immunocytes to the site of tissue injury, such as monocytes/macrophages and neutrophils^21^. Previous reports showed that a decrease in MCP-1 levels, induced by high-dose methylprednisolone treatment, attenuated macrophage accumulation in the ischemic brain of rat^36^. Application of mNOX-E36, a pharmacological inhibitor of MCP-1, could inhibit hepatic macrophage infiltration in CCl_4_-induced acute liver injury and MCD diet-induced steatohepatitis^22^. In addition, MCP-1 plays a major role in recruiting neutrophils^37^. In our study, we also observed that there was more macrophage infiltration in injured liver than that in the vehicle control. Owing to the powerful role of MCP-1 in the recruitment of immunocytes in inflammatory diseases, in the present study, we selected MCP-1 as a representative of inflammatory molecules to delineate the mechanism of hepatocellular cytokine regulation. We believe that the increased MCP-1 induced by MIF in hepatocytes might help recruit macrophages in the initial phase of acute liver injury. These results imply, from another perspective, that hepatocytes are involved in the pathological process of acute liver injury through the secretion of inflammatory molecules.

Accumulating evidence indicates that the expression of MIF is generally increased in a variety of diseases associated with inflammation^16^,^38^, and decreasing the expression of MIF may ameliorate the degree of liver inflammation^3^,^39^. In the present study, the expression of MIF was evidently increased in injured liver induced by CCl_4_, which corroborates reports that MIF expression was upregulated in injured mouse liver tissue treated with an ethanol-containing liquid diet for 4 days, or in rat liver that suffered ischemia and reperfusion injury^3^,^39^. In addition, elevated MIF expression was also observed in clinical disease such as autoimmune liver disease, acute myocardial infarction, and kidney disease^16^,^40^,^41^. As an important inflammatory cytokine, MIF is rapidly released in response to various stimulations^39^,^40^. In our study, hepatocyte-secreted MIF protein was significantly increased at 15 min after LPS treatment, which was not delayed compared with the increase in MIF mRNA. This may be because, in contrast to most of the cytokines, MIF is prestored in intracellular pools, and secreted immediately by a non-conventional protein-secretion pathway before de novo protein synthesis^10^. Supported by the observation in rat liver ischemia and reperfusion-injury model, the increase in MIF mRNA in liver tissue was parallel with that of serum MIF protein content at 30 min after liver ischemia and reperfusion^39^. These results emphasize the importance of the increase in MIF levels in the incipient stage of different diseases. However, the biological function attributed to hepatic MIF remains unclear, and should be studied in further research.

Several studies have revealed that MIF can, either directly or indirectly, promote the expression of many other inflammatory molecules^19^,^39^,^40^. It was reported that MIF-deficient mice were protected from ethanol-induced increase in MCP-1 and MIP-1 expression^3^. Veillat *et al.*^13^ demonstrated that MIF could directly stimulate protein expression of IL-8, MCP-1, and VEGF in human ectopic endometrial stromal cells in a time- and dose-dependent manner^13^. Furthermore, MIF application can induce dose-dependent increase in IL-6, IL-8, and PGE2 release from chondrocytes^12^. Consistent with previous researches, we found that the increased MIF levels in injured hepatocytes could directly act on the hepatocytes via receptors in an autocrine manner, to promote the expression and release of inflammatory chemokine MCP-1. Further, administration of MIF inhibitor decreased MCP-1 expression in injured liver effectively. Significant increase in MCP-1 expression by MIF stimulation was also reported in podocytes^19^. However, in addition to MCP-1, other inflammatory molecules that are regulated by MIF in injured hepatocytes and participate in liver inflammation, are still to be investigated. Since MIF is a soluble cytokine, the receptor’s assistance is necessary for MIF’s biological function. Results presented here showed that the increase in MCP-1 expression induced by MIF, LPS, or CCl_4_ could be effectively blocked by the deletion of CD74 and CD44, which are the classical receptor and coreceptor of MIF, respectively. Notably, the effect of CD74 and CD44-specific siRNAs in LPS-treated hepatocytes was much weaker than that in rMIF-treated hepatocytes. LPS affects a lot of inflammatory cytokines expression directly through inflammatory signaling pathways, such as NF-κB and MAPK signaling pathway^42^,^43^. Although our results showed that MIF induced by LPS can mediate MCP-1 expression, but LPS itself has an ability to increase MCP-1 expression as well^44^. That reminds us that MIF is not the unique factor in regulation of MCP-1 expression in the injured hepatocytes treated by LPS.

MIF can influence a variety of signaling pathways via CD74 and CD44, and further affects the biological function of cells. It is reported that MIF regulates pulmonary vasoconstriction through PKC, p38, and ERK signaling pathway^29^. In addition, MIF acts through the AMPK signaling pathway to induce cellular resistance to glucose deprivation, ischemia, hypoxia, and oxidative stress^30^. Moreover, MIF induces matrix metallopeptidase-9 expression in mouse macrophage mainly via the ERK MAPK pathway; matrix metallopeptidase-2 production induced by MIF in rheumatoid synovial fibroblasts requires the activation of PKC^45^,^46^. Based on this, pharmacological inhibitors were employed to identify the molecular mechanism underlying the regulation of MCP-1 expression in hepatocytes. The MCP-1 increase induced by MIF decreased significantly after pretreatment with a specific inhibitor of p38. However, inhibition of PKC, ERK, or AMPK had no effect on MCP-1 expression. These results implied p38 as the critical mediator of MCP-1 expression induced by MIF. CD44 can trigger Syk activation in neutrophils, which promotes phosphorylation of p38^47^, and its knockdown suppresses both mRNA and protein levels of c-Src and its downstream MAPK pathway^48^. Therefore, we speculated that MIF might amplify the p38 signaling pathway through CD44, and then promote MCP-1 expression in hepatocytes. By Western blot analysis, we showed that MIF alone could strengthen the phosphorylation level of p38, while CD74 or CD44 deficiency markedly inhibited p38 activation. In addition, ISO-1 could alleviate the hepatic phosphorylation level of p38 *in vivo*. These results illustrated that MIF regulated MCP-1 expression in hepatocytes through CD74/CD44 and the p38 MAPK pathway.

In summary, our findings present evidence that MIF secreted by injured hepatocytes is an important regulator of MCP-1 expression in hepatocytes. The earlier expressed MIF amplifies the p38 MAPK signaling pathway through its receptors CD74 and CD44, and then drives the increased expression of MCP-1 in injured hepatocytes. These results provide compelling new information on the role of MIF in liver injury and open up new perspectives on mechanistic research on hepatic injury.

## Methods

### Materials

Recomniant mouse MIF protein were acquired from R&D systems (Minneapolis, USA). LPS and Collagenase IV were obtained from Sigma (Missouri, USA). SB203480 (p38 MAPK inhibitor), PD98059 (ERK inhibitor), staurosporine (PKC inhibitor) and compound C (AMPK inhibitor) were purchased from Tocris Bioscience (Bristol, UK). PCR reagents were from Applied Biosystems (Life Technologies, Foster City, CA). ISO-1 was from Abcam (Cambridge, MA).The other common reagents were from Sigma (St. Louis, MO).

### Mouse models of acute liver injury

Mouse models of acute liver injury were performed by injection of CCl_4_^49^. The adult ICR mice were treated with 1 μL/g body weight of CCl_4_ diluted (1:9 v/v) in olive oil (OO) by intraperitoneal injections. The mice were sacrificed at 24 hours, 48 hours and 72 hours after CCl_4_ treatment (n = 6 per group). All animal work was approved by the Ethics Committee of Capital Medical University and in accordance with the approved guidelines (approval number: AEEI-2014-131).

### Mouse primary hepatocytes preparation

Primary hepatocytes were isolated from adult mice as described previously^50^. To isolate primary murine hepatocytes, anesthetized and heparinized mice were subjected to a midline laparotomy and cannulation of the portal vein followed by liver perfusion with an EGTA-chelating perfusion buffer (EGTA: 190 mg, glucose: 900 mg, HEPES: 10 mL of 1 M stock solution, KCl: 400 mg, Na_2_HPO_4_-12H_2_O: 305 mg, NaCl: 8 g, NaH_2_PO_4_-2H_2_O: 88 mg, and NaHCO_3_: 350 mg, made up to 1 L with dH_2_O). After perfusion with 0.4% collagenase buffer (CaCl_2_-2H_2_O: 560 mg, HEPES: 10 mL of 1 M stock solution, KCl: 400 mg, Na_2_HPO_4_-12H_2_O: 305 mg, NaCl: 8 g, NaH_2_PO_4_-2H_2_O: 88 mg, NaHCO_3_: 350 mg, and collagenase IV: 400 mg, made up to 1 L with dH_2_O), livers were minced and cells dispersed in culture medium; hepatocytes and nonparenchymal cells were separated using low-speed centrifugation and 40% percoll density gradient centrifugation. Isolated mouse hepatocytes (2 × 10^5^ /well) were cultured in William’s Medium E (Gibco, Life Technologies, Foster City, CA) with 10% FBS on 24-well collagen-coated plate at 37 °C with 5% CO_2_ for 4 hours. Hepatocytes were incubated in the presence or absence of LPS (100 ng/mL) or rMIF (100 ng/mL). After 6 hours culture, the cells were used for Immunofluorescence assay, real-time RT-PCR and ELISA assay.

### Cells culture

Mouse liver cell lines AML-12 (ATCC CRL-2254) was cultured in DMEM/F12 (Gibco, Life Technologies, Foster City, CA) supplemented with 10% FBS, 100 U/mL penicillin and 100 mg/mL streptomycin, at 37 °C, in a humidified 5% CO_2_ atmosphere.

### RNA Interference

The siRNA sequences specifically targeting mouse MIF, CD74 or CD44 were synthesized by Ambion (1320003, Ambion, Austin, TX). 40–50% confluent AML-12 cells were prepared. Transient transfection of siRNAs (40 nmol/L) was performed using Invitrogen Lipofectamine RNAiMAX (Invitrogen, Grand Island, NY), as recommended by the manufacturer. Control cells were treated with 40 nmol/L RNAi Negative Control Duplexes (scrambled siRNAs). After 48 hours, cells were used to perform further assay.

### Real-time RT-PCR

Total RNA was extracted from liver tissue or cells using an RNeasy kit (Qiagen, Hilden, Germany). Real-time RT-PCR was performed in an ABI Prism 7300 sequence detecting system (Applied Biosystems, Foster City, CA), as described previously^51^. Primers used for real-time RT-PCR were as follows: mouse MIF sense, 5′-GCCAGAGGGGTTTCTGTCG-3′ and antisense, 5′-GTTCGTGCCGCTAAAGTCA-3′; mouse MCP-1 sense, 5′-TCTGGGCCTGCTGTTCACA-3′ and antisense, 5′-GGATCATCTTGCTGGTGAATGA-3′; mouse CD74 sense, 5′-CACCACTGCTTACTTCCTGTACCA-3′ and antisense, 5′-GCAGGTTCTGGGAGGTGATG-3′; mouse CD44 sense, 5′-CAGATTCCAGAATGGCTCATCA-3′ and antisense, 5′-GATGCAGACGGCAAGAATCA-3′; and 18S rRNA: sense, 5′-GTAACCCGTTGAACCCCATT-3′ and antisense, 5′-CCATCCAATCGGTAGTAGC-3′.

### Immunohistochemistry

Liver tissues were fixed in 4% paraformaldehyde for 24 hours and embedded in paraffin. 5 μm sections were performed immunostaining by using a commercially available kit (ZSGB-BIO, Beijing, China)^52^. Briefly, the slides were dewaxed in xylene, rehydrated in graded alcohol and rinsed in phosphate buffer solution (PBS) for 5 min. Antigen retrieval was done by immersing the slides in citrate buffer solution (pH 6.0) and microwave heated for 20 minutes. Endogenous peroxidase activity was quenched by incubating the slides with 3% hydrogen peroxide for 10 minutes. Following incubation with the 2% bovine serum albumin (Roche, Switzerland), the sections were incubated with anti-MIF primary antibody (1:200, Santa Cruz Biotechnology, Santa Cruz, CA) overnight at 4 °C. Goat anti-rabbit secondary antibody conjugated with IgG-HRP was applied for 30 minutes. Color development was done with the peroxidase substrate 3-amino-9-ethylcarbazole. Between steps, slides were rinsed with PBS. Counterstaining of nucleus was done with hematoxylin solution. The mean optical density of IHC images was measured by Image-Pro Plus software (Media Cybernetics, MD).

### Immunofluorescence

Liver samples were fixed in 4% paraformaldehyde and embedded in Tissue Tek OCT compound (Electron Microscopy Sciences, Japan). Five micrometers of frozen section were used for immunofluorescence. After blocked with 3% BSA (Roche, Switzerland), the sections were incubated with F4/80 antibody (1:250, Santa Cruz Biotechnology, Santa Cruz, CA) followed by secondary antibody conjugated with FITC (1:100, Jackson Immuno-Research, West Grove, PA). At last, nuclei were stained with DAPI. The number of F4/80+ cells were measured by ImageJ software (an open source Java image processing program, http://imagej.net/). Mouse primary hepatocytes or AML-12 cells were fixed with 4% paraformaldehyde for 30 minutes and permeabilized with 0.5% Triton X-100 (Amresco, OH) for 15 minutes. After blocked with 3% BSA, they were incubated with MIF antibody (1:200, Santa Cruz Biotechnology, Santa Cruz, CA) followed by secondary antibody conjugated with FITC (1:100, Jackson Immuno-Research, West Grove, PA). At last, nuclei were stained with DAPI.

### Western blot analysis

Western blot analysis was carried out with standard procedures and followed primary antibodies against MIF (1:500; Santa Cruz Biotechnology, Santa Cruz, CA), p38 (1:1000; Cell Signaling, Beverly, MA) or phospho-p38 (1:1000; Cell Signaling, Beverly, MA). IRDyeTM 800-conjugated Goat anti-rabbit IgG or goat anti-mouse IgG (1:10,000, LI-COR Biosciences, Lincoln, NE) was applied appropriately as secondary antibodies. Protein expression was visualized and quantified by the LI-COR Odyssey® Imaging System and Odyssey® software (LI-COR Biosciences, Lincoln, NE), respectively. Results were normalized relative to GAPDH (rabbit antibody, 1:1000; Sigma, St. Louis, MO) or β-Tubulin (1:1000; Cell Signaling, Beverly, MA) expression to correct for variations in protein loading and transfer.

### ELISA

The cells culture supernatants were collected and used for ELISA assay followed by the specification (Cusabio, Wuhan, China).

### Statistical Analysis

Data are presented as means ± standard error of the mean (SEM). Differences between groups were evaluated using a two-sided Student’s t-test. *P* < 0.05 was considered significant. All results were confirmed in at least three independent experiments.

## Additional Information

**How to cite this article**: Xie, J. *et al.* Macrophage Migration Inhibitor Factor Upregulates MCP-1 Expression in an Autocrine Manner in Hepatocytes during Acute Mouse Liver Injury. *Sci. Rep.* **6**, 27665; doi: 10.1038/srep27665 (2016).

## Supplementary Material

Supplementary Information
