# Supplementary material for: Macrophage Migration Inhibitor Factor Upregulates MCP-1 Expression in an Autocrine Manner in Hepatocytes during Acute Mouse Liver Injury
Source: Sci Rep. 2016 Jun 8;6:27665. doi: 10.1038/srep27665 (PMC4897699; doi:10.1038/srep27665)
Supplement: Supplementary Information [file srep27665-s1.pdf]

Macrophage Migration Inhibitor Factor Upregulates MCP-1 Expression in an Autocrine Manner in Hepatocytes during Acute Mouse Liver Injury

Jieshi Xie, Le Yang, Lei Tian, Weiyang Li, Lin Yang and Liying Li*

Supplementary information

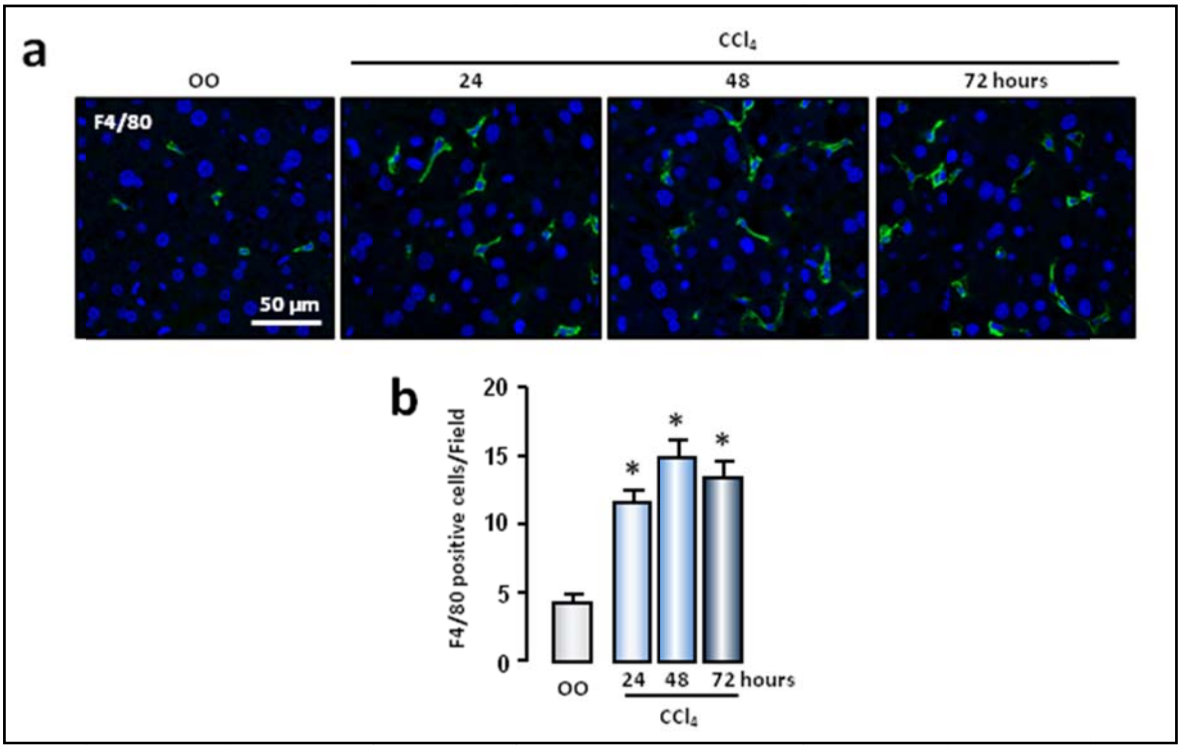

Supplementary Figure 1. Macrophages infiltration was increased in CCl_4 induced liver injury. (a) Representative images of immunofluorescence by confocal microscopy to track macrophages (F4/80+, green) in the CCl_4-treated liver, DAPI was used to visualize nuclei (blue). (b) Quantification of macrophages (F4/80+, green) with Image-Pro Plus software. Scale bars, 50 µm. Data are presented as the means ± SEM. *$P < 0.05$ *vs* control group (n=6, per group).

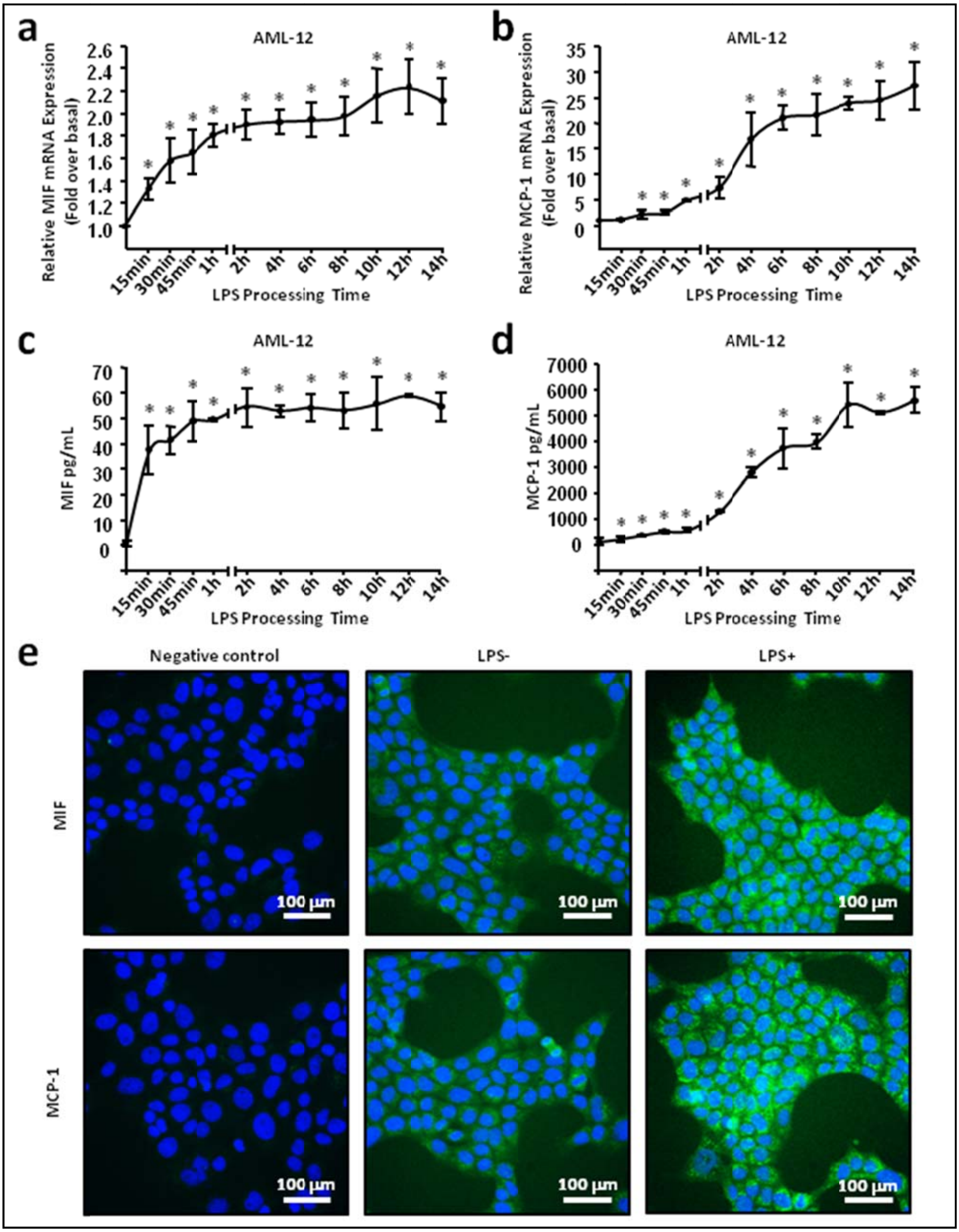
a
AML-12
Relative MIF mRNA Expression (Fold over basal)
LPS Processing Time
b
AML-12
Relative MCP-1 mRNA Expression (Fold over basal)
LPS Processing Time
c
AML-12
MIF pg/mL
LPS Processing Time
d
AML-12
MCP-1 pg/mL
LPS Processing Time
e
Negative control
LPS-
LPS+
MIF
MCP-1
100 μm

Supplementary Figure 2. LPS induced increase of MIF earlier than MCP-1 in AML-12 cells. AML-12 cells were treated with 100 ng/mL LPS and collected at the described time points, the relative mRNA (a, b) expression and protein secretion (c, d) of MIF and MCP-1 were examined by real-time RT-PCR and ELISA. (e) The representative images of immunofluorescence for MIF or MCP-1 (green) in AML-12 cells, as visualized by immunocytochemical analysis. Cells were co-stained with DAPI to identify nuclei (blue). Scale bars, 100 μm. All results were confirmed in three independent experiments at least. *P < 0.05 vs untreated control cells.

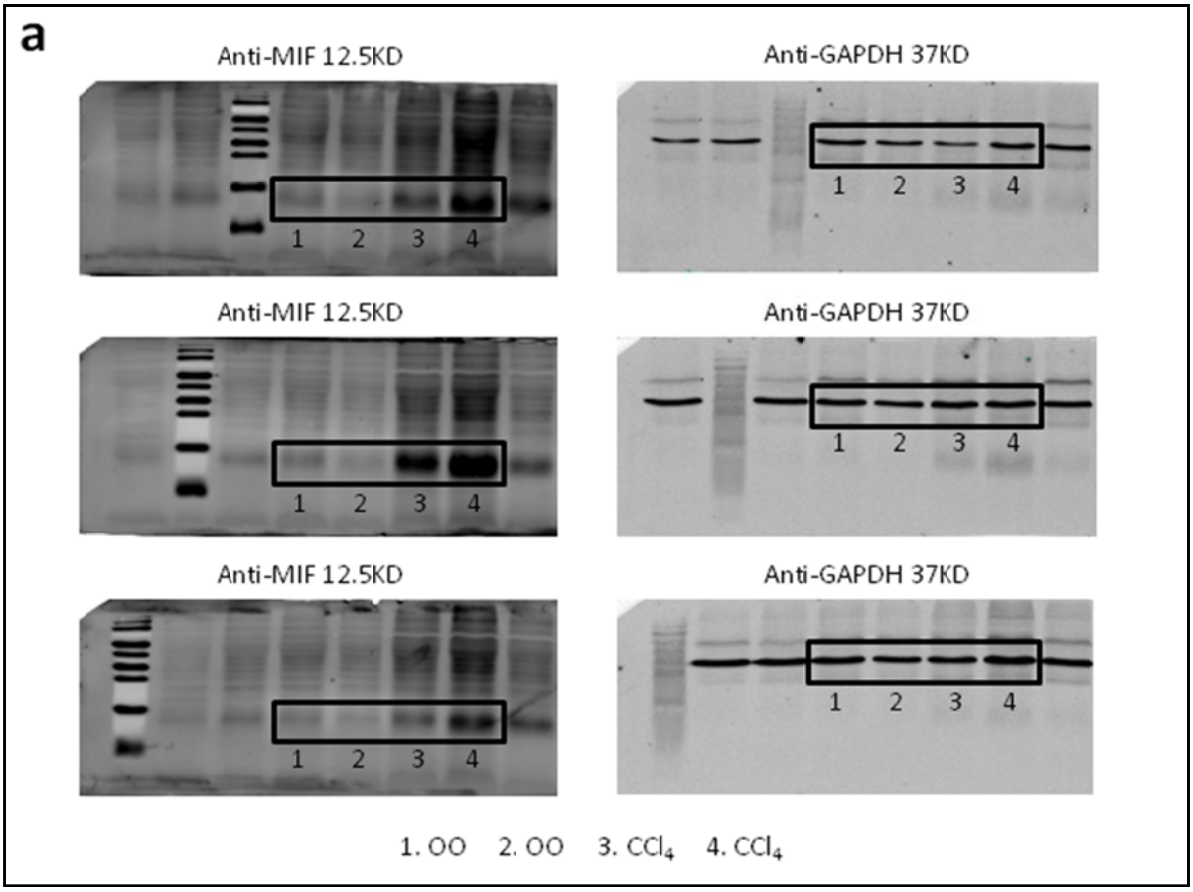

Supplementary Figure 3. The full-length blots of Figure 1b.

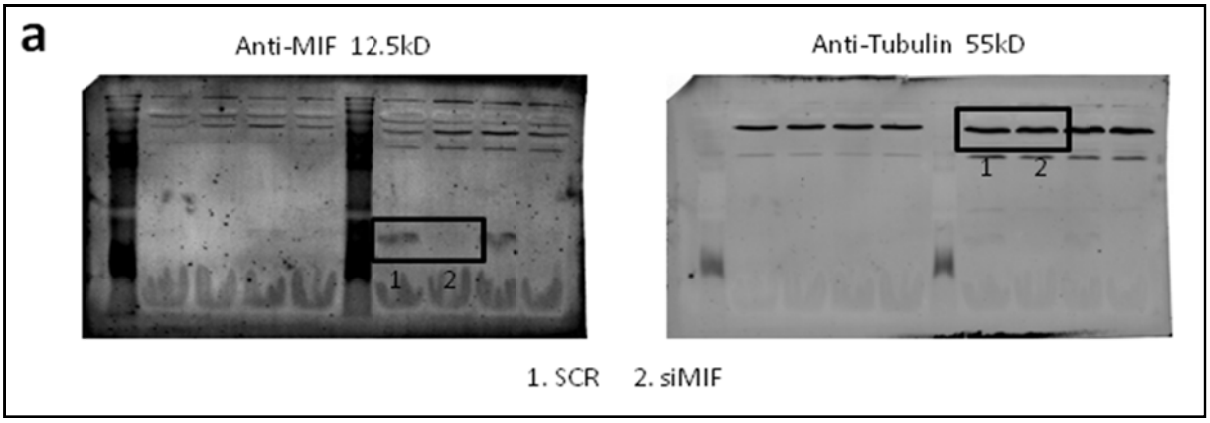

Supplementary Figure 4. The full-length blots of Figure 3b.

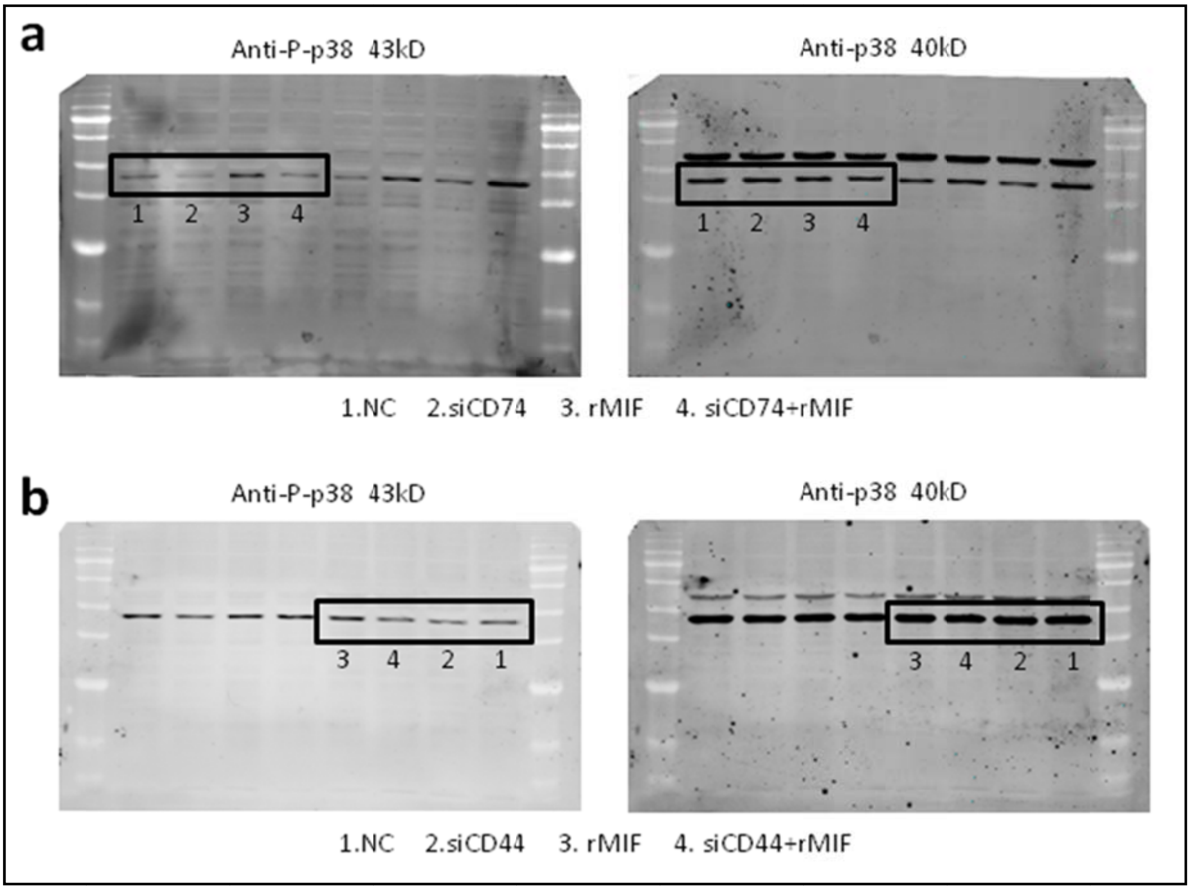

Supplementary Figure 5. The full-length blots of Figure 5. (a) The full-length blots of Figure 5c. (b) The full-length blots of Figure 5d.

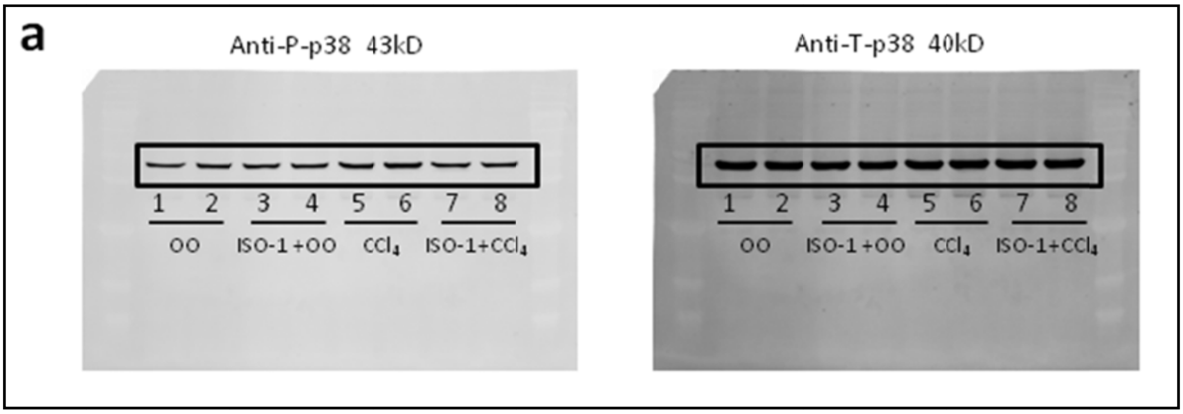

Supplementary Figure 6. The full-length blots of Figure 6c.
